# Protease inhibitor monotherapy is associated with a higher level of monocyte activation, bacterial translocation and inflammation

**DOI:** 10.7448/IAS.17.1.19246

**Published:** 2014-09-29

**Authors:** Berta Torres, Alberto C Guardo, Lorna Leal, Agathe Leon, Constanza Lucero, Míriam J Alvarez-Martinez, Miguel J Martinez, Jordi Vila, María Martínez-Rebollar, Ana González-Cordón, Josep M Gatell, Montserrat Plana, Felipe García

**Affiliations:** 1 Infectious Diseases Unit, Hospital Clínic, IDIBAPS, University of Barcelona, Barcelona, Spain; 2 Retrovirology and Viral Immunopathology Laboratory, AIDS Research Group, IDIBAPS, Hospital Clínic, University of Barcelona, Barcelona, Spain; 3 Department of Clinical Microbiology, Hospital Clínic, CRESIB, University of Barcelona, Barcelona, Spain

**Keywords:** protease inhibitor, monotherapy, immune activation, very low-level viremia, microbial translocation, monocyte

## Abstract

**Introduction:**

Monotherapy with protease-inhibitors (MPI) may be an alternative to cART for HIV treatment. We assessed the impact of this strategy on immune activation, bacterial translocation and inflammation.

**Methods:**

We performed a cross-sectional study comparing patients on successful MPI (*n*=40) with patients on cART (*n*=20). Activation, senescence, exhaustion and differentiation stage in CD4+ and CD8+ T lymphocyte subsets, markers of monocyte activation, microbial translocation, inflammation, coagulation and low-level viremia were assessed.

**Results:**

CD4+ or CD8+ T lymphocyte subset parameters were not significantly different between both groups. Conversely, as compared with triple cART, MPI patients showed a higher proportion of activated monocytes (CD14+ CD16−CD163+ cells, *p*=0.031), soluble markers of monocyte activation (sCD14 *p*=0.004, sCD163 *p*=0.002), microbial translocation (lipopolysaccharide (LPS)-binding protein; LBP *p*=0.07), inflammation (IL-6 *p*=0.04) and low-level viremia (*p*=0.035). In a multivariate model, a higher level of CD14+ CD16−CD163+ cells and sCD14, and presence of very low-level viremia were independently associated with MPI. Monocyte activation was independently associated with markers of inflammation (IL-6, *p*=0.006), microbial translocation (LBP, *p*=0.01) and low-level viremia (*p*=0.01).

**Conclusions:**

Patients on MPI showed a higher level of monocyte activation than patients on standard therapy. Microbial translocation and low-level viremia were associated with the high level of monocyte activation observed in patients on MPI. The long-term clinical consequences of these findings should be assessed.

## Introduction

The introduction of HIV antiretroviral treatment has completely changed the spectrum of the illness that has become, in the past years, a chronic condition in developed countries. Prompt diagnosis has also permitted to treat patients earlier and the proportion of patients that are first diagnosed with a low number of CD4+ T lymphocytes or with an opportunistic infection has sharply decreased. With stable patients on treatment and living longer, the morbidity associated with medication and cost of treatment has notably increased. Strategies for simplifying treatment and lowering the cost have started to be implemented [1]. Monotherapy with ritonavir-boosted protease inhibitor has been proposed as one of these alternatives.

Several clinical trials have assessed the virological effectiveness of simplification of a successful standard protease inhibitor containing regimen to a protease inhibitor monotherapy. The MONET and the MONOI studies compared ritonavir-boosted darunavir in monotherapy versus the standard triple therapy treatment. Both demonstrated non-inferiority in the per-protocol analysis [2,3]. In the same way, the OK study showed similar rates of viral suppression between the boosted lopinavir monotherapy and the triple therapy group [4].

On the contrary, lower rates of virological suppression were seen in the MONARK study in naïve patients who started monotherapy with boosted lopinavir as compared with standard triple therapy containing regimen [5]. Finally, in the MOST study virologically suppressed patients for at least six months with standard cART were randomized to switch to protease inhibitor monotherapy or to continue with triple therapy. This study was prematurely stopped due to high rates of virologic failure [6].

Overall, monotherapy seems an effective strategy in patients previously suppressed with no prior virologic failure. However, more episodes of transient viremia elevation have been reported in the monotherapy groups included in the studies [3,7], although a higher incidence of PI mutations was not observed [3].

Efficacy in the above-cited clinical trials was determined by viral load (VL) suppression, but no other variables were taken into account. Concerns about the possibility of a higher immune activation in patients on monotherapy, or the possibility of lower penetration in the central nervous system were raised. Few studies were performed in an attempt to answer this question. A retrospective study of the samples in the MONET trial showed no differences in high-sensitive C-reactive protein (hs-CRP) or interleukin-6 (IL-6) between patients in monotherapy or triple therapy [8]. Suppression in CSF has also been studied in patients on boosted lopinavir monotherapy versus triple therapy, with similar findings in both groups [9]. However, some case reports in the literature describe patients on PI monotherapy and with suppressed VL in plasma who showed detectable VL in CSF [10,11]. Moreover, in the MONOI trial, two patients in the monotherapy group that presented neurological symptoms showed VL escape in the CSF when a lumbar puncture was performed [3].

We hypothesized that patients on monotherapy could have a higher level of immune activation than patients on cART and, therefore, were at higher risk of suffering long-term clinical consequences (i.e. non-AIDS events). Here, we perform a study to compare the impact of successful monotherapy, either with boosted darunavir or boosted lopinavir, on immune activation, microbial translocation and other inflammatory markers.

## Patients and methods

### Patients

Forty patients on antiretroviral treatment who had successfully simplified to monotherapy either with ritonavir-boosted darunavir or ritonavir-boosted lopinavir (MPI group) were recruited between September 2011 and September 2012 in the outpatient HIV clinic in the Hospital Clinic, Barcelona. Patients had to fulfil the following inclusion criteria: age over 18 years, antiretroviral treatment with monotherapy for at least the previous 48 weeks and VL <37 copies/ml in the blood tests performed in the previous 48 weeks. Patients fulfilling the inclusion criteria and willing to participate signed informed consent and an extraction of 60 ml of blood was performed. Twenty patients on triple therapy with a PI-containing regimen (ritonavir-boosted darunavir or lopinavir) (cART group) were recruited as a comparison group. To participate in the study, written informed consent was obtained from all individuals, and the study protocol was evaluated and approved by the Hospital Ethical Committee.

### VL measurement

Plasma HIV-RNA was measured using Versant HIV-1 RNA v3.0 (Siemens, Barcelona, Spain), which has a lower limit of detection and a lower limit of quantification of 37 copies/ml. Patients with a plasma VL that was detectable but below the limit of quantification (<37 copies/ml) were classified as patients with very low-level viremia (VLLV), and those patients with VL reported as not detected were classified as undetectable [13].

### Cell samples

**Figure 1:**
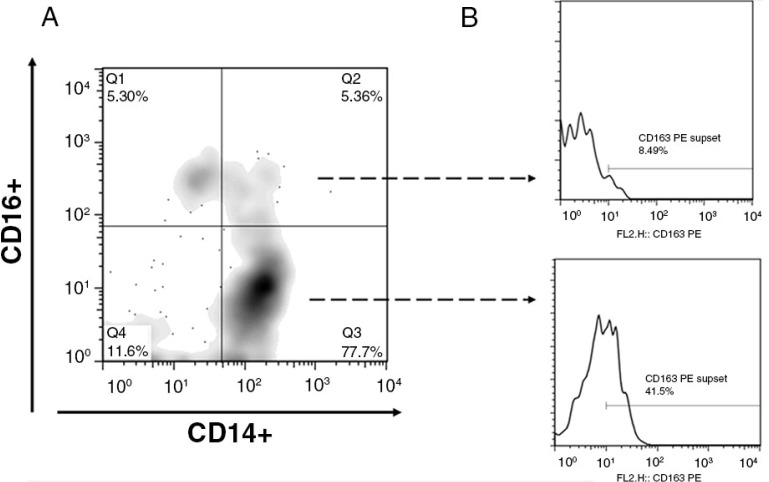
Identification and analysis of monocyte subpopulations. (A) Gated monocytes were subdivided into monocyte subpopulations on the basis of CD14 and CD16 staining characteristics. Subpopulations are defined as CD14+ CD16+ (quadrant 1), CD14++ CD16+ (quadrant 2), CD14++ CD16− (quadrant 3), and CD14-CD16− (quadrant 4). The percentage of cells for each population is depicted in the outer most corner. (B) CD163 activation marker was measured according to the flow cytometry gating strategy indicated above in CD14+CD16– (Q3) and CD14+ CD16+ (Q1+Q2).

All analyses were done in freshly isolated peripheral blood mononuclear cells (PBMCs). EDTA-anticoagulated blood was obtained by venipuncture; PBMCs were immediately isolated by density gradient centrifugation using Ficoll-Hypaque (Sigma Chemical Co., St. Louis, MO). We used a comprehensive approach of simultaneous measurement of different immunological parameters [12] in CD4+ and CD8+ T lymphocyte: activation (using CD38 and HLADR markers), senescence (using CD28 and CD57 marker), exhaustion (using PD-1 marker), co-receptor expression (CCR5, CXCR4), differentiation stage (using CD45RA and CD45RO markers) and monocyte activation (using CD14, CD16 and CD163 markers). The stained cells were analyzed on a FACSCalibur (Becton Dickinson, San Jose, CA) cytometer. Flow cytometry gating strategy for monocytes is shown in Figure 1. Data were analyzed using FlowJo Software (Tree Star).

### Soluble markers of monocyte activation, microbial translocation, inflammation and coagulation markers

Serum was initially frozen at a temperature of −80°C. Soluble markers of monocyte activation [soluble CD14 (sCD14) and soluble CD163 (sCD163)], markers of host response to lipopolysaccharide (LPS) [LPS-binding protein (LBP), endotoxin-core IgM antibody (EndoCAb)], inflammation [high-sensitive C-reactive protein (hs-CRP), interleukin-6 (IL-6), tumour necrosis factor-alpha (TNF-alpha)] and pro-coagulation (D-dimer) were assessed.

Soluble CD14 was determined with Quantikine ELISA (R&D systems; limit of detection 125 pg/ml; intra-assay variability is 4.8–6.4%); and soluble CD163 was determined by Macro163 ELISA (IQ products; limit of detection 0.23 ng/ml; intra-assay variability 3–6%) according to manufacturer's instructions. Human LBP and endotoxin-core antibodies were determined by ELISA (Hycult biotech; limit of detection 4.4 ng/ml and 0.05 MMU/ml, respectively). High-sensitive CRP was determined by an immune turbidimetric method (CardioPhase, Siemens Healthcare Diagnostics). A result over 0.5 mg/dl was considered positive. IL-6 and TNF-alpha were determined by ELISA (Diasource Immunoassays, Louvain-la-Neuve, Belgium). Results over 5 and 10 pg/ml, respectively, were considered positive. D-dimer was used as a pro-coagulation marker. It was measured with a turbidimetric method (Innovance, Siemens Diagnostics, Marburg, Germany) in a BCS automated coagulation system XP (Siemens Diagnostics). The sensitivity of the technique allows for the detection of levels as low as 10 ng/ml. The normal cut-off is 500 ng/ml.

### Statistical analysis

Characteristics of the study population and the different immunological parameters, microbial translocation and inflammatory markers were recorded as median [interquartile range], and comparisons were made using *t*-test or the non-parametric tests Mann–Whitney *U*-test. Correlations between quantitative parameters were explored using the Spearman's rho test. Logistic regression was used to analyze the factors independently associated with the type of therapy (MPI vs. cART). Multiple regression was used to analyze the factors independently associated with monocyte activation. All statistical analyses were performed using the SPSS software version 20 (SPSS Inc., Chicago, IL, USA). All *p*-values were two-tailed, and were considered significant when lower than 0.05.

## Results

### Clinical characteristics

No differences in age or gender were observed between the two groups (Table 1). Patients with HCV co-infection were equally distributed between groups. Patients on monotherapy had a longer time since HIV diagnosis and had been on antiretroviral treatment longer. The accumulated time on a PI-containing regimen was longer for the patients on monotherapy but without reaching statistical significant difference. The median time on ritonavir-boosted protease inhibitor monotherapy was 37 months (IQR: 13–51 months). CD4+ T lymphocyte count at start of antiretroviral treatment was lower in the MPI group; however, the CD4+ T lymphocyte count at the time of inclusion in the study was similar between the two groups with a median >500 cells/mm^3^ in both groups. No differences in plasma VL at the start of antiretroviral treatment were seen between groups. All patients had VL <37 copies in the last control performed before the inclusion in the study, but in the blood test performed the day of inclusion, four patients had detectable VL (two patients in the MPI group with values 50 and 52 copies/ml, and two in the cART group, with values of 156 and 39 copies/ml).

**Table 1:** Clinical characteristics of patients

Characteristic	MPI group (*n*=40)	IP-containing triple therapy (*n*=20)	*P*
Age	49 (45–57)	44 (40–52)	0.71
Sex (female). no,%	11 (27.5)	4 (20)	0.753
Time since HIV diagnosis (months)	184.3 (146.3–223.5)	96.9 (64.9–222.3)	0.048
Time on ART (months)	167.5 (102.7–183.0)	67.2 (39.8–156.3)	0.006
Time on a PI-containing regimen (months)	91.4 (48.2–123.7)	49.8 (22.0–123.1)	0.074
VL <37 copies. no, %	38 (95)	18 (90)	0.595
VL at start of treatment (Log)	5,14 (4,06–5,49)	4,60 (4,08–5,49)	0.71
CD4 cell count (cells/mm^3^) at inclusion	566 (390–830)	568 (429–706)	0.660
CD4 cell count (cells/mm^3^) at start of treatment	192 (65–281)	299 (119–381)	0.03
CD4 cell count *Nadir*	145 (48–227)	243 (118–319)	0.16
HCV co-infection. no, %	9 (22.5)	5 (25)	1
Type of PI on the past 12 months (ABT/DRV)	26/14	12/8	0.780

VL=viral load; ABT=lopinavir/ritonavir; DRV=darunavir/ritonavir; PI=protease inhibitor; HCV=hepatitis C virus; ART=antiretroviral treatment; MPI=Monotherapy with ritonavir-boosted protease inhibitor. cART: triple therapy with a PI-containing regimen (ritonavir-boosted darunavir or lopinavir).

Data are in median [IQR], unless otherwise indicated.

### Comparison of immunological parameters between MPI and cART groups

**Figure 2:**
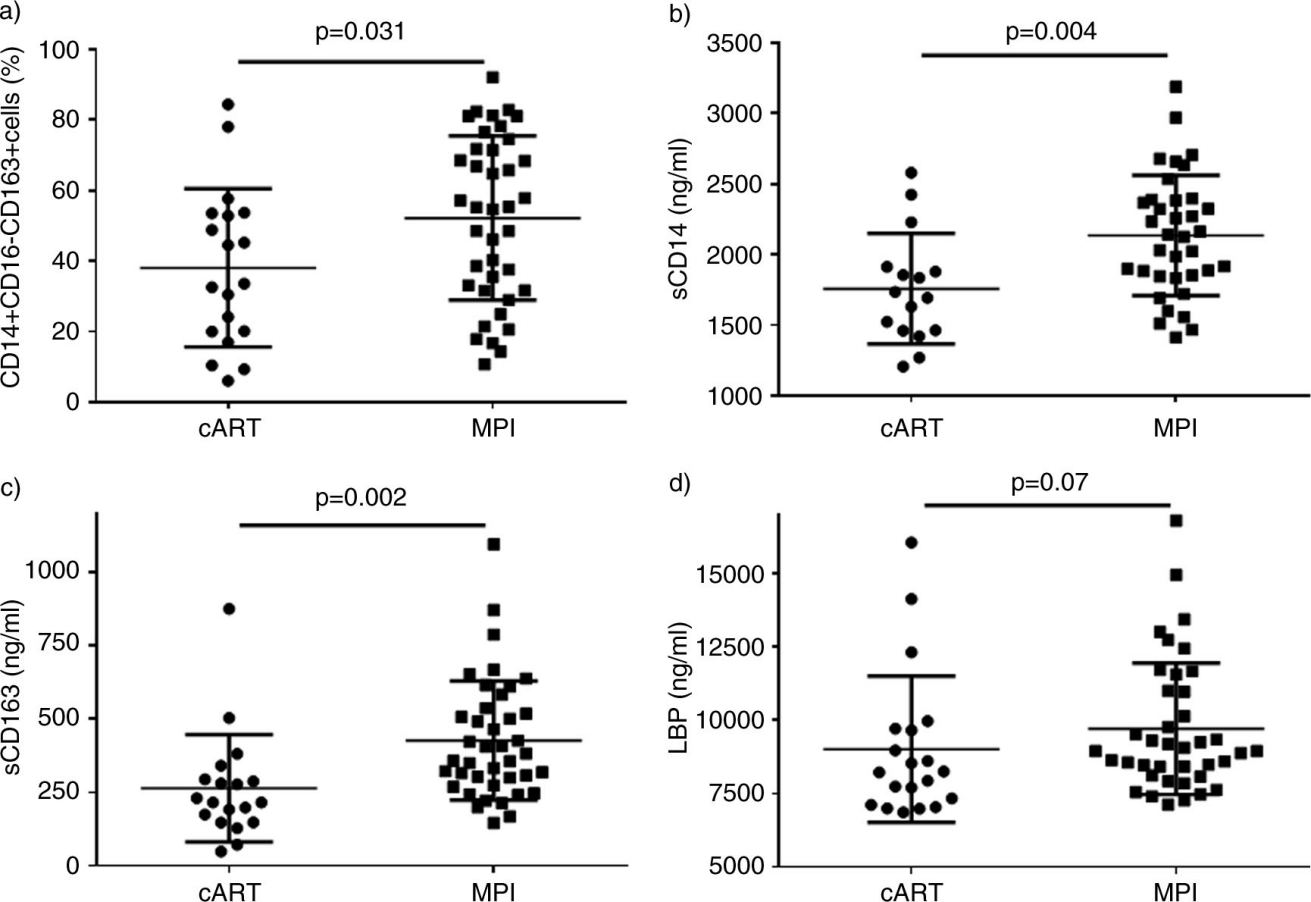
Comparison of the levels of monocyte activation and markers of microbial translocation between patients on triple protease inhibitor regimen vs. patients on protease inhibitor monotherapy.

No differences were observed in markers of T lymphocyte activation, senescence, exhaustion, co-receptor expression or differentiation stages between both groups (see Table 2). Conversely, as compared with cART, MPI patients showed a higher proportion of activated monocytes (CD14+CD16+CD163+ cells 9.63% vs. 7.53%, *p*=0.033; CD14+ CD16− CD163+ cells 55.17% vs. 33.57%, *p*=0.031 (Figure 2A); and CD14+CD16+ cells 26.85% vs. 19.79%, *p*=0.029, MPI vs. cART groups)

**Table 2:** Lymphocyte and monocyte subpopulations

Cell subset category	Subpopulations (%)	PI Monotherapy (*n*=40)	IP-containing triple therapy (*n*=20)	*p*
Activation of CD4 and CD8 T cells	CD4 DR+38+	2.23 (1.59–3.73)	2.62 (1.57–3.91)	0.5483
	CD8 DR+38+	8.3 (5.2–12.62)	8.33 (6.01–14.73)	0.8525
Senescence	CD4 28−57+	1.73 (0.64–3.55)	2.63 (0.83–5.18)	0.3944
	CD8 28−57+	28.09 (18.19–39.97)	31.71 (26.87–43.95)	0.2363
Exhaustion	CD4 PD1+	20.54 (14.02–30.33)	18.65 (16.58–27.56)	0.7212
	CD8 PD1+	22.72 (18.38–31.35)	25.30 (21.29–29.52)	0.4605
Co-receptors	CD4 CCR5+	25.22 (12.59–38.26)	24.63 (15.13–29.36)	0.7030
	CD8 CCR5+	14.66 (7.42–26.77)	16.85 (6.59–23.24)	0.8839
Differentiation stage	CD4 RA+RO−	31.27 (24.41–48.34)	31.88 (21.13–40.84)	0.9032
	CD8 RA+RO−	46.40 (36.09–54.47)	45.60 (36.38–54.45)	0.9677
	CD4 RA-RO+	57.51 (40.77–69.30)	57.81 (35.94–63.45)	0.5008
	CD8 RA-RO+	40.06 (28.49–52.23)	39.69 (25.29–47.85)	0.5647
Monocyte activation	CD14+16+163+	9.63 (3.65–15.62)	7.53 (3.23–9.09)	0.033
	CD14+16−163+	55.17 (31.58–71.76)	33.57 (20.00–53.53)	0.031
	CD14+16+163−	13.51 (10.81–21.71)	12.04 (9.88–15.97)	0.1518
	CD14+16+	26.85 (18.99–41.06)	19.79 (14.94–25.88)	0.029

### Comparison of soluble markers of monocyte activation, markers of microbial translocation, inflammation, pro-coagulation and VLLV between MPI and cART groups

Levels of soluble markers of monocyte activation were higher in MPI vs. cART patients [sCD14: 2133 vs. 1714 ng/ml, *p*=0.004 (Figure 2B); sCD163: 369 vs. 215 ng/ml, *p*=0.002 (Figure 2C)] (Table 3). LBP levels, but not EndoCab were also higher in MPI patients [LBP: 8948 vs. 8237 ng/ml, *p*=0.07 (Figure 2D)]. Regarding inflammatory markers, only IL-6 levels were significantly higher in the MPI group as compared with cART (IL-6: 34.5 vs. 8 pg/ml, *p*=0.04). D-dimer levels were similar between both groups. Given that patients on monotherapy had a significantly longer time of known HIV infection and a lower CD4+ T lymphocyte count before starting cART and these variables could mean a bias to compare both groups, we repeated the analysis controlling both variables, excluding those patients on monotherapy with longer follow-up (more than 12 years, lower IQR of MPI group) and lower CD4+ T lymphocyte count previous to treatment (below 200 cells/mm^3^, median value of MPI group). The differences in monocyte activation, microbial translocation and inflammatory markers between both groups were confirmed (data not shown).

**Table 3:** Markers of bacterial translocation, inflammation, pro-coagulation and very low-level viremia

	PI Monotherapy (*n*=40)	IP-containing triple therapy (*n*=20)	*p*
Bacterial translocation			
LBP (ng/ml)	8948 (8115–10,999)	8237 (7171–9693)	0.07
sCD14 (ng/ml)	2133.18 (1847.13–2387.14)	1714.36 (1460.53–1904.20)	0.004
EndoCAb (MMU/ml)	68 (44–99)	74 (34–105)	0.47
sCD163 (ng/ml)	368 (279.76–530.90)	215.38 (147.18–292.23)	0.002
Inflammation			
hs-CRP (mg/dl)	0.2 (0.08–0.46)	0.18 (0.09–0.33)	0.72
IL-6 (pg/ml)	34.5 (18–258)	8 (8–27.5)	0.04
TNFα (pg/ml)	5 (4–7)	4.5 (4–7)	0.27
Pro-coagulation			
D-dimer (ng/ml)	201 (90–320)	176 (111–269)	0.23
Viremia			
Detection of VLLV. *n* (%)	15/38 (39.4)	2/18 (11)	0.03

LBP=Lipopolysaccharide-binding protein; sCD14=soluble CD14; EndoCAb=endotoxin core IgM antibody; sCD163=soluble CD163; hs-CRP=high sensitive C-reactive protein; IL-6=interleukin-6; VLLV=very low-level viremia. Data are in median (IQR) unless otherwise indicated.

Finally, we analyzed the presence of VLLV, defined as viremia below limit of detection (37 copies) but qualitatively detectable. Fifteen out of 38 (39%) patients in the monotherapy group presented positive signal under the limit detection versus two out of 18 (11%) in the triple therapy one (*p*=0.035)****.

A multivariate analysis was performed to assess the factors independently associated with the type of therapy (MPI vs. cART). Variables with statistically significant differences in univariate analysis were included in the model. A higher level of CD14+ CD16− CD163+ cells (*p*=0.023) and sCD14 (*p*=0.013), and presence of VLLV (*p*=0.027) were independently associated with MPI. We repeated the multivariate model controlling for CD4+ T lymphocyte at the start of the antiretroviral treatment, CD4+ T lymphocyte at inclusion, nadir CD4+ T lymphocyte and time of HIV infection. The results showed that CD14+ CD16− CD163+ cells and sCD14, but not VLLV, remained independently associated with MPI.

### Correlations among monocyte activation levels, markers of microbial translocation and inflammatory markers

Patients with higher levels of monocyte activation showed the higher levels of microbial translocation and inflammatory markers. The levels of CD14+CD16+CD163+ cells were correlated with sCD14 (rho=0.29, *p*=0.039), sCD163 (rho=0.26, *p*=0.047), hsPCR (rho=0.29, *p*=0.02); the levels of CD14+CD16+ cells were correlated with IL-6 (rho=0.70, *p*=0.003) and TNF-alpha (rho=0.36, *p*=0.006); and the levels of sCD14 with LBP (rho=0.54, *p*<0.0001), hsPCR (rho=0.29, *p*=0.038) and TNF-alpha (rho=0.41, *p*=0.003). Markers of monocyte activation were not correlated with CD4+ T cell at start of antiretroviral treatment or nadir CD4+ T lymphocyte.

In a multivariate analysis (using in the model as dependent variable either CD14+CD16+CD163+ cells or CD14+CD16+ cells), monocyte activation was independently associated with markers of inflammation (IL-6, *p*=0.006) and low-level viremia (*p*=0.01). In addition, in a multivariate model using as dependent variable sCD14, this soluble marker of monocyte activation was independently associated with the marker of microbial translocation LBP (*p*=0.028) and low-level viremia (*p*=0.05).

In addition to sCD14, the marker of microbial translocation LBP was directly correlated with sCD163 (rho=0.28, *p*=0.034) and inflammatory markers hs-PCR (rho=0.45, *p*<0.0001) and TNF-alpha (rho=0.37, *p*=0.004).

**Figure 3:**
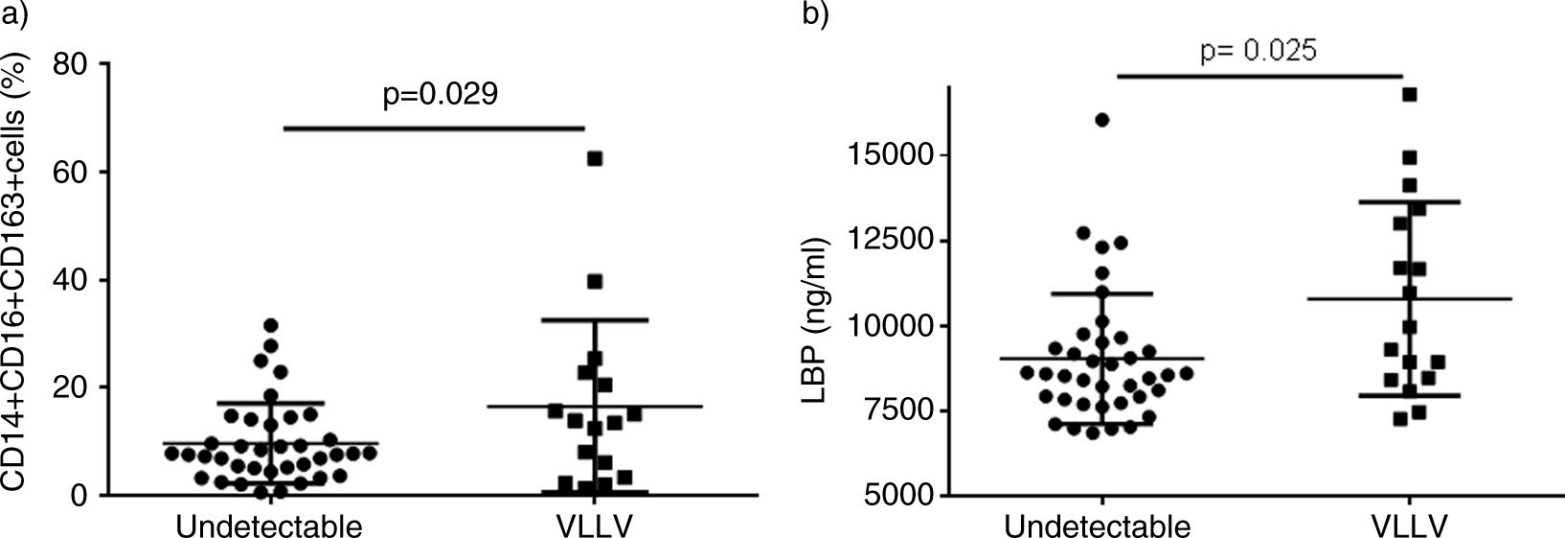
Comparison of the levels of monocyte activation and markers of microbial translocation between patients with undetectable viral load and patients with very low-level of viremia.

Finally, as compared with patients with undetectable level of VL, patients with VLLV showed a higher percentage of CD14+CD16+CD163+ cells [median 13.65, IQR (4.02–22.17) vs. 7.65 (4.21–13.31), *p*=0.029] and higher levels of LBP [median 9964 ng/ml, IQR(8452–13,213) vs. 8574 (7820–9548), *p*=0.025] (Figure 3).

## Discussion

The results of our study show a higher level of monocyte activation in patients on successful monotherapy with a boosted PI as compared with protease inhibitor standard regimen. The level of monocyte activation was associated with markers of host response to microbial translocation, inflammation and low-level viremia. These data might suggest that microbial translocation and VLLV drive the high level of monocyte activation observed in these patients on protease inhibitor monotherapy.

The first objective of our study was to assess if the simplification to a successful monotherapy had an impact on activation of the immune system. Although no differences were observed in the percentage of CD4+ and CD8+ T lymphocytes subpopulations (measuring activation, senescence, exhaustion and differentiation stage), patients on monotherapy with a boosted PI showed a higher level of monocyte activation as measured by the expression of CD16 and CD163 in CD14 cells, and the levels in plasma of soluble CD14 and CD163. Recent studies describe higher percentage of markers of monocyte activation in HIV viremic patients and, also in HIV-treated patients who, despite effective antiretroviral treatment, still express higher monocyte activation than age matched uninfected patients [14,15]. The marker CD16 is known to be expressed in more mature monocytes with a distinct pattern of cytokine production and they are considered to be proinflammatory monocytes [16]. CD163 is a monocyte/macrophage haemoglobin scavenger receptor involved in the anti-inflammatory response of monocytes [17]. CD163 is predominantly expressed in CD14+ CD16− monocytes in the general population and in those who are HIV positive [17,18]. Surface expression of CD163 also accounts for activation and can be co-expressed altogether with CD16 [19]. Both CD163 and CD14 are shed upon monocyte activation and the soluble form can be detected in plasma. The increase of all these markers of monocyte activation has been associated with clinical end-points. The frequency of inflammatory CD16+ monocytes is associated with risk of coronary artery progression [20]. Soluble CD14 was reported to be an independent predictor of mortality in HIV [21] and has been related to the increase in the yearly rate of carotid intima-media thickness [22], whereas soluble CD163 has been associated with increased risk of coronary artery inflammation and atherosclerosis and with subclinical atherosclerosis [23]. In addition, a study that assessed neurocognitive impairment in virologically suppressed HIV patients found higher levels of soluble CD163 in patients with a higher global deficit score, suggesting persistent monocyte activation in neuropsychological impaired patients [24]. All these data suggest that the long-term consequences of elevated monocyte activation in patients on PI monotherapy should be further assessed in longitudinal studies.

The second objective of the study was to investigate the factors associated with monocyte activation in this cohort. We found that patients with higher monocyte activation had a higher level of IL-6, LBP and low-level of viremia. These data might suggest that microbial translocation and VLLV drive the monocyte activation in patients on protease inhibitor monotherapy. We found that IL-6 was significantly higher in the monotherapy group. It has been reported that increases in concentrations of interleukin-6 are strongly associated with all-cause mortality [25]. If the higher level of IL-6 would be associated with a worse long-term clinical outcome in these patients deserves further investigation.

Early gut mucosal destruction in HIV infection results in microbial translocation and higher microbial products in the circulation that are supposed to be partially responsible of HIV-associated immune activation that persists despite the introduction of the antiretroviral treatment [26]. A recent study has shown an increase in mucosal macrophages in HIV naïve patients due to enhanced trafficking of blood monocytes in the gut but with lower phagocytic activity [27]. In addition, products of microbial translocation, such as LPS, bind to LBP and cause monocyte activation via toll-like receptor 4 [28]. Higher microbial translocation is then closely related to monocyte activation and has been related to disease progression [29], neurocognitive impairment [30], subclinical atherosclerosis [22] and other pathogenic conditions as non-Hodgkin lymphoma [31] in HIV patients. Our data support these findings, since we observed not only that microbial translocation was associated with monocyte activation, but that both microbial translocation and monocyte activation were associated with an increase of inflammatory markers (hsPCR, IL-6 and TNF-alpha).

Finally, we observed that a higher number of patients included in the MPI group presented with VLLV. The importance of VLLV has been a recent issue of interest. Some studies have related VLLV to a higher risk of virologic failure, and have suggested that low-level viral replication is a cause of VLLV [32,33]. In our study, when we compared patients with VLLV with patients with undetectable VL, we observed a higher percentage of activated monocytes (CD14+CD16+CD163+) and levels of LBP in the first group, suggesting a relation between VLLV and monocyte activation. Traditionally, monocytes were considered to be non-permissive for HIV infection. However, replication competent virus can be detected following activation of these cells [34]. A study that characterized HIV-1 RNA in treated patients with low-level viremia (<48 copies) and examined the sources of residual plasma viremia compared to that expressed in CD4+ T lymphocytes and in CD14+ CD16+ monocytes observed that plasma sequences were more related to that sequenced in activated monocytes, suggesting that residual viremia found in cART-suppressed patients could have its origin from cells from the myeloid lineage [35]. We could hypothesize that monotherapy with a boosted PI regimen is less able to control viral replication in reservoirs as monocyte subsets, leading to higher monocyte activation, inflammation and microbial translocation.

We are aware that our study has other limitations. First of all, it is a transversal study and a low number of patients are included. Second, regarding baseline characteristics, the CD4+ T lymphocyte count at the start of treatment was lower in the MPI group, although no significant differences were observed in VL, nadir of CD4+ or CD4+ T lymphocyte count at the time of inclusion. In fact, it has been reported that clinically important CD4+ T lymphocyte count responses are likely to be better defined in terms of absolute postcART CD4+ T lymphocyte counts, rather than change from baseline [36]. In addition, we have recently reported that differences in CD4+ T lymphocyte gain with different cART regimen are not immunologically meaningful [37]. Moreover, we have repeated the analysis controlling by time of known HIV infection and CD4+ T lymphocyte count previous to cART and the results were confirmed. Finally, VLLV as defined by plasma VL that was detectable but below the limit of quantification could not be a good measurement of the reservoir or residual viremia. In fact, other studies [38,39] did not find an increase of the level of persistent viremia as measured by Roche Amplicor HIV-1 RNA assay with a quantification limit of three copies/ml or by single-copy assay. Apart from the technique used for the measurement, the main difference with our study is that in these studies residual viremia was assessed during the first 48 weeks of simplification, while in our study the median time on monotherapy was three years and all of the patients were on monotherapy for at least the previous 48 weeks before the inclusion and had a VL <37 copies/ml in all the blood tests performed in the previous 48 weeks. We know that the best option for assessing persistent viremia was to measure residual viremia directly; regretfully, we did not have enough sample availability to measure it by other techniques.

In summary, the higher monocyte activation observed in the monotherapy group raises concern about this strategy and the possible association with a higher mortality and long-term cardiovascular and neurocognitive deleterious effects. Larger clinical trials should be performed in order to confirm these results.
